# Pseudohyperkalemia Associated With Leukemia

**DOI:** 10.7759/cureus.23978

**Published:** 2022-04-09

**Authors:** Biraj Shrestha, Swarup Sharma Rijal, Arpan Pokhrel, Anish Paudel, Krantikiran Baral, Bidhya Poudel, Sijan Basnet, Anthony Donato

**Affiliations:** 1 Internal Medicine, Reading Tower Health, Reading, USA; 2 Internal Medicine, St. Francis Hospital, Illinois, USA; 3 Internal Medicine, The Reading Hospital and Medical Center, Wyomissing, USA

**Keywords:** iatrogenic complications, hyperkalemia, point-of-care testing, leukemia, pseudo hyperkalemia

## Abstract

Elevated potassium levels can be a life-threatening emergency. We describe a case of falsely elevated serum potassium level in a patient with leukemia, which was suspected to be falsely elevated because the patient was asymptomatic with a normal electrocardiogram (EKG). Common reasons behind such a discrepancy in leukemia patients are the use of a tourniquet before collection, use of vacuum/pneumatic tubes for transportation, prolonged periods of incubation, use of heparin for sample collection, and processing of samples via centrifugation. Since the process is related to the method of collection and processing, we recommend using rapid point of care testing in such cases to differentiate between false and true potassium elevation, as it is a well-validated tool. Moreover, there is a good correlation between potassium measured with the blood gas, point of care, and central laboratory analyzers when the concentration of potassium is above 3 mEq/L.

## Introduction

Hyperkalemia is a common potentially life-threatening condition. When found in an asymptomatic leukemia patient with no electrocardiographic changes, it can lead to an unnecessary study, emergency referral, and treatments. Unnecessary treatment can be particularly detrimental and life-threatening if it leads to arrhythmias due to hypokalemia from overcorrection. Falsely elevated potassium levels are often termed “pseudohyperkalemia.” Pseudohyperkalemia can arise in several clinical scenarios, including in patients with severe leukocytosis or patients with hemolyzed samples due to sampling or laboratory processing issues. Early recognition of this condition can prevent unnecessary treatments and iatrogenic complications. We present a case of a 73-year-old man with pro-lymphocytic leukemia with pseudohyperkalemia [1].

## Case presentation

**Figure 1:**
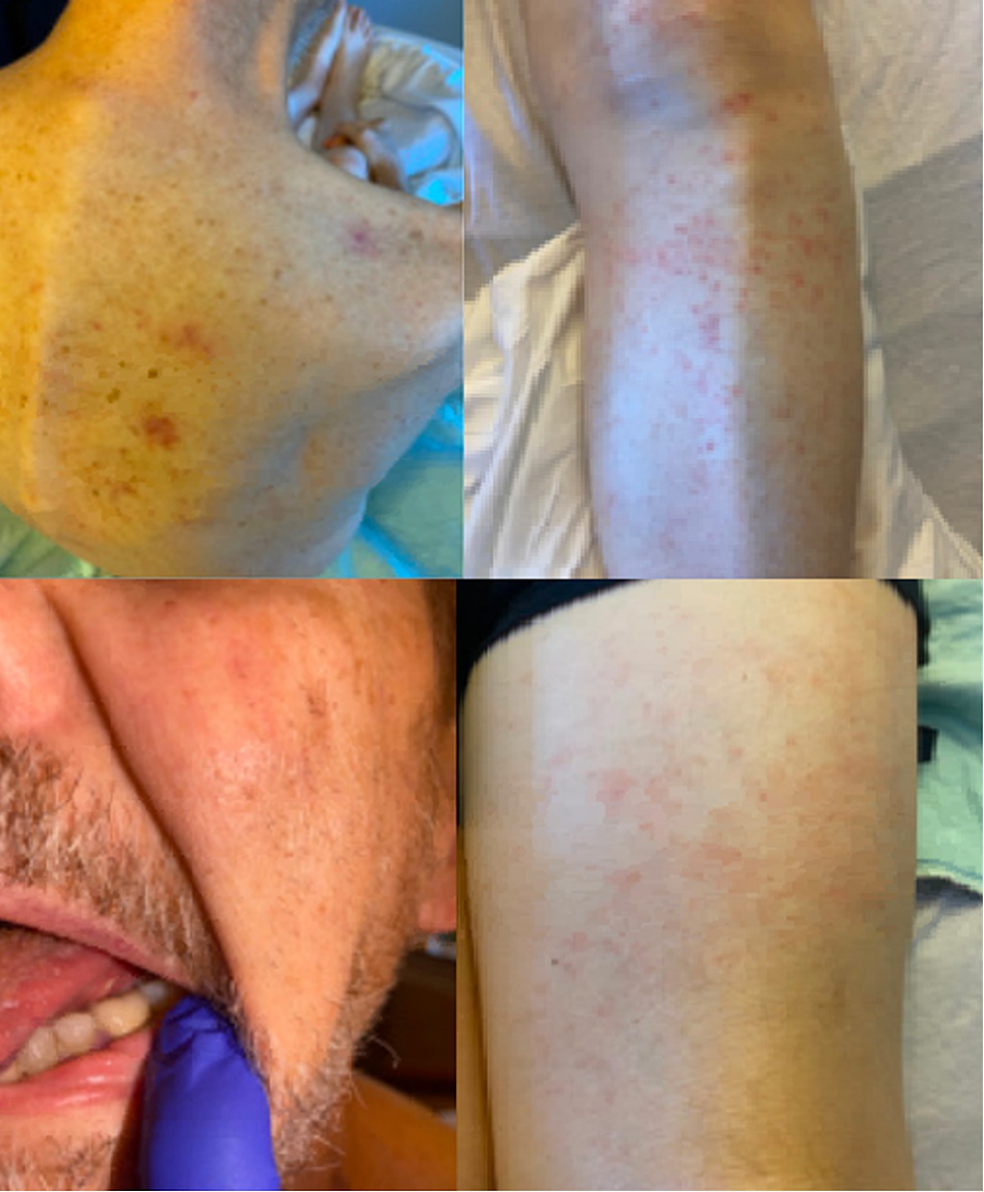
Picture of the back, thigh, and shin of the patient showing nonpalpable, non-branchable, non-tender, and erythematous punctate rash. Picture of the tongue showing an aphthous ulcer on the ventral surface of the tongue.

A 73-year-old man with a medical history of recently diagnosed B-cell prolymphocytic leukemia one month ago and started on acalabrutinib presented to the emergency department (ED) with a fever of 101.4°F and progressive odynophagia. His other home medications included allopurinol, lisinopril, and aspirin. Since starting acalabrutinib, he also complained of feeling shortness of breath with exertion and progressive fatigue. He also reported rashes on his chest, abdomen, and legs (Figure 1). On examination, he was alert and oriented. His blood pressure was 110/60 mmHg, pulse was 102 beats/minute, and was saturating at 98% room air. He had multiple non-tender and firm lymph nodes in his axillary, cervical, and inguinal regions, hepatosplenomegaly, multiple aphthous ulcers in his mouth (Figure 1), and a non-tender, erythematous, diffuse non-blanching rash most prominent on bilateral shins, chest, and back.

Laboratory workup showed a white blood cell (WBC) count of 67.6 x 103/microliter (reference range: 4.8 - 10.8 x 103/μL) but a neutrophil count of 790/μL (reference range: 2-8 x 103/μL), platelet count of 67 x 103/μL (reference range: 130 - 400 x 103/μL), and hemoglobin of 8.4 grams/dL (reference range: 14.0 - 17.5 g/dL). His admission creatinine was 1.33 mg/dL (reference range: 0.60 - 1.30 mg/dL) and serum potassium was 5.1 mmol/L (reference range: 3.5 - 5.1 mmol/L). Given the presence of rash, fever, odynophagia, and neutropenia, there was high suspicion for infection, so labs were sent for infectious workup, which included blood cultures, and cytomegalovirus (CMV) polymerase chain reaction (PCR). 

He was admitted for neutropenic fever and started on intravenous vancomycin, cefepime, fluconazole. Because of neutropenia, he received one injection of filgrastim. On the second day of hospitalization, his hemoglobin decreased to 6.1 g/dL without any obvious source of bleeding. Hemolytic workup showed lactate dehydrogenase 13 IU/L (reference range: 140 - 271 IU/L), reticulocyte count: 0.2% (reference range: 0.5 - 2.0 %), negative direct Coombs test, and haptoglobin 88 mg/dL (reference range 44 - 215 mg/dL). His disseminated intravascular panel showed fibrin split products: <10 ug/mL (reference range: <10 ug/mL), international normalized ration(INR): 1.8 (reference range: 0.9-1.1 ), activated partial thromboplastin time: 30.9 seconds (reference range: 23.5-32.9 seconds ), fibrinogen: 150 mg/dL (reference range: 193-488 mg/dL), D-dimer: 0.45 ug/mL (reference range: <0.50 ug/ml). He received two units of packed red blood cells. His post-transfusion lab showed hemoglobin 8.2 g/dL (reference range: 14.0 - 17.5 g/dL) and potassium was 5.2 mmol/L (reference range: 3.5 - 5.1 mmol/L). As per oncology recommendations, he underwent a bone marrow biopsy that showed marrow involvement by prolymphocytic lymphoid cells.

**Figure 2:**
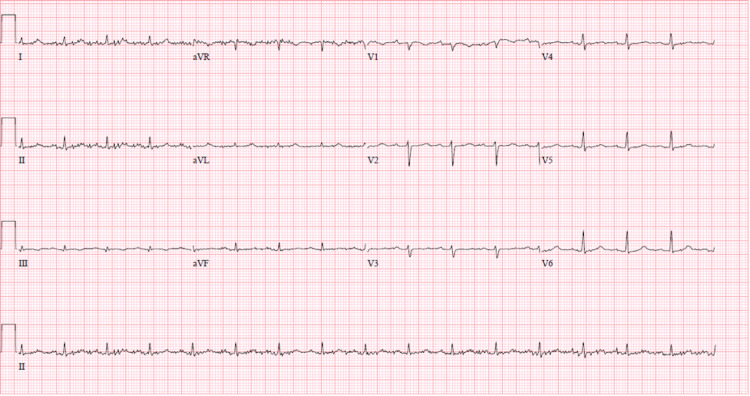
Sinus rhythm 100 beats per minute, normal axis, no ST or T wave changes

He improved with empiric symptomatic treatment and discontinuation of his acalabrutinib. All cultures and viral titers were negative. The morning labs on the day of discharge, which was the sixth day of admission, showed potassium of 6.7 mmol/L (reference range: 5.0-5.2 mmol/L). Labs were collected in a lithium heparin tube and analyzed within 1.5 hours with an AEO Beckman Coulter blood analyzer (Brea, California). The specimen was reported not hemolyzed. Other pertinent laboratory studies included a creatinine of 1.09 mg/dL (reference range: 0.60 - 1.30 mg/dL), calcium of 7.1 mg/dL (reference range: 8.6 - 10.3 mg/dL), albumin of 2.7 g/dL (reference range: 3.5-5.7 g/dL), and WBC count of 89.1 x 103/ μL (reference range: 4.8 - 10.8 x 103/μL), with 98% lymphocytes (reference range: 14 - 48 %). He was asymptomatic. Electrocardiogram (EKG) didn’t show any of the changes expected in hyperkalemia (Figure 2). There was a concern for tumor lysis syndrome because of the low calcium and high WBC count. However, his uric acid was 2.7 mg/dL (reference range: 4.4 - 7.6 mg/dL) and phosphorus was 5.7 mg/dL (reference range: 2.5 - 5.0 mg/dL).

Given his history of leukemia and asymptomatic presentation, we did point-of-care potassium immediately, which came back normal at 4.1 mmol/L (reference range: 3.5 - 4.9 mmol/L). Because the point-of-care potassium was normal, the patient was diagnosed to have pseudohyperkalemia, and we did not start any treatment. The patient was discharged home with outpatient follow-up with hematology/oncology. Subsequent readmission after discharge also showed the patient had potassium of greater than 10.0 mmol/L while point-of-care potassium repeated at the same time was 4.3 mmol/L (reference range: 3.5 - 4.9 mmol/L).

## Discussion

There are two major non-iatrogenic mechanisms of in vivo true hyperkalemia, which are lysis of cells, which results in the secondary release of potassium, and decreased urinary excretion of potassium due to poor renal function [1].

Patients with hyperkalemia typically manifest muscle weakness, paralysis, and distinct electrical changes on the EKG. The classic EKG changes begin with peaked T-waves, which can progress to a widened PR interval, widened QRS interval, loss of the P wave, and depressed ST segment, which ultimately can result in a sinusoidal waveform. These EKG changes may or may not occur in order. Hence, if any of these changes are present, it should increase the suspicion for the cardiac involvement of hyperkalemia [2]. In situations where there is a slow rise in potassium, for example, in a patient with end-stage renal disease, it may result in the development of cardiac tolerance to the persistent high potassium in the system, which could present with unremarkable EKG findings. Hence results of EKG in such patients must be interpreted with caution [3-4].

Pseudohyperkalemia is an in-vitro phenomenon in which there is a false elevation in measured potassium concentration. This usually occurs due to the movement of potassium out of the cell during or after a blood specimen has been collected [5-6]. It should be suspected in stable and asymptomatic patients without significant CKD with no electrical changes on their EKG. Several factors that have been thought to play a role in its development are the use of a tourniquet before collection, the use of vacuum/pneumatic tubes for specimen transportation, prolonged periods of specimen incubation, and processing of samples via centrifugation, especially in patients with a high WBC count. These factors have been thought to cause cell lysis in vitro, which results in false potassium elevation. This phenomenon occurs even when the potassium concentration is measured in a heparinized tube, where clotting is less likely to occur. It is also thought that there may be leakage of potassium from fragile white blood cells in patients with leukemia, leading to falsely elevated potassium. This is theorized to occur due to a lack of energy to maintain sodium/ potassium adenosine triphosphatase activity for the collected large number of white cells in-vitro, resulting in the release of potassium contributing to false hyperkalemia [2,5,7-8]. Another proposed mechanism is that the heparin in the collection tube causes damage to the membranes of the brittle malignant cells [8]. This has been shown in several studies by findings of higher plasma potassium levels in the simultaneously collected samples of heparinized tubes compared to non-heparinized tubes [8]. There is also a directly proportional relationship between potassium concentration and the amount of heparin in the tubes [8]. Hence, high potassium in asymptomatic leukemia patients with no EKG changes requires careful evaluation before treatment is begun. The recommendation is to confirm potassium level by arterial blood gas analysis, as this is quick and reliable, especially in patients with a WBC count > 100 x 103/mm^3 ^[7].

Potassium concentration is typically measured in core laboratory analyzers by using an ion-selective electrode (ISE). It measures the potassium concentration by using a voltmeter, which detects the electric potential of an ion dissolved in a solution [9]. We can use both serum and plasma to measure potassium. There is always a higher concentration of potassium in serum when compared with plasma because platelets release potassium during the process of clotting [1].

In point-of-care testing, direct, undiluted unfractionated whole blood is used to measure potassium by electrochemical methods [1]. Investigators have found that there is a good correlation between electrolytes measured with the blood gas, point of care, and central laboratory analyzers when the concentrations of electrolytes are above 3 mEq/L [1]. Chacko et al. found that the differences between the point-of-care potassium and core laboratory analyzer potassium were large and clinically significant when the concentration of potassium measured was below 3 mEq/L, with the point-of-care measurements being up to 1 mEq/L lower than central laboratory analyzer [10]. Hawkins demonstrated that we missed over 33% of hypokalemic cases when we used point-of-care measurement to determine the concentration of potassium [11]. Studies have shown that there is a negligible variability of potassium values measured between the blood gas and core laboratory analyzers [12-13].

## Conclusions

Pseudohyperkalemia can be missed and result in life-threatening iatrogenic hypokalemia. Therefore, it should be suspected in stable and asymptomatic patients without significant CKD with no electrical changes on their EKG despite a very high reported level of potassium. Common reasons behind such a discrepancy in leukemia patients are the use of a tourniquet before collection, use of vacuum/pneumatic tubes for transportation, prolonged periods of incubation, use of heparin for sample collection, and processing of samples via centrifugation, which have been thought to cause cell lysis in vitro, resulting in false potassium elevation. Hence, we recommend using point-of-care testing or arterial blood gas analysis if there is a question of pseudohyperkalemia, especially in asymptomatic hyperkalemia patients with leukocytosis. The logic behind this is that point-of-care testing or arterial blood gas analysis is reliable and involves immediate processing and gentle handling of the blood sample leading to the decreased traumatic release of potassium and false hyperkalemia.
